# The TIR Domain Containing Locus of *Enterococcus faecalis* Is Predominant among Urinary Tract Infection Isolates and Downregulates Host Inflammatory Response

**DOI:** 10.1155/2014/918143

**Published:** 2014-07-24

**Authors:** Thomas Daniel Kraemer, Orlando Daniel Quintanar Haro, Eugen Domann, Trinad Chakraborty, Svetlin Tchatalbachev

**Affiliations:** ^1^Institute of Medical Microbiology, Justus Liebig University, Schubertstraße 81, 35392 Giessen, Germany; ^2^Iztacala Superior Studies Faculty, National Autonomous University of Mexico, Avenida de los Barrios 1, Los Reyes Iztacala, 54090 Tlalnepantla, MEX, Mexico

## Abstract

Based on Toll/interleukin-1 receptor (TIR) domain structure homology, we detected a previously uncharacterized gene encoding for a TIR domain containing protein (Tcp) in the genome of *Enterococcus faecalis*. We assigned this gene the name *tcpF* (as in Tcp of *E. faecalis*). Screening of *E. faecalis* samples revealed that *tcpF* is more common in isolates from urinary tract infections (UTIs) than in human faecal flora. *tcpF* alleles showed moderate single nucleotide polymorphism (SNP) among UTI isolates. Infection of mouse RAW264.7 macrophages with a *tcpF* knock-out mutant led to elevated cytokine response compared to the isogenic wild type *E. faecalis* strain. *In silico* analysis predicted significant tertiary structure homology to the TIR domain of human TLR1 (TLR1-TIR). When transiently expressed in cultured eukaryotic cells, TcpF caused suppression of TLR2-dependent NF-*κ*B activation suggesting for TcpF a role as a factor in *E. faecalis* that benefits colonization by modulating the host's immune responses.

## 1. Introduction

Signaling via Toll-like receptors (TLRs) requires the homo- or heterodimerization of the receptors. The process is initiated by their extracellular leucine-rich repeats (LRRs) regions and leads to dimerization of the receptor's cytoplasmic Toll/interleukin-1 receptor (TIR) domains which form a TIR-TIR structure [1]. This TIR-TIR structure provides the site of association with proteins of the TIR domain-containing adapter family [2, 3]. Activation of TLR pathways leads to the release of inflammatory cytokines like tumor necrosis factor *α* (TNF-*α*) and interleukin 6 (IL6), type I interferons (IFNs), and chemokines, which in turn control the recruitment of inflammatory cells to the infected tissues [4].

The central role of TLR signaling in innate immunity makes it a target for bacterial induced immune subversion. In 2006 the TIR-like protein, TlpA, from* Salmonella enterica*, was reported as a novel prokaryotic modulator of NF-*κ*B activity and interleukin 1 beta (IL-1*β*) secretion that contributes to serovar Enteritidis virulence by interfering with TLR4 and MyD88 signaling [5]. Similar observations were also made with the TIR domain containing proteins TcpC of* Escherichia coli* and TcpB of* Brucella melitensis *[6]. TcpC was found to impair the signaling of TLRs and the secretion of proinflammatory cytokines IL6 and TNF-*α*. Furthermore,* tcpC* was associated with the severity of urinary tract infections (UTIs) [6]. It was demonstrated that the TIR domain of TcpC was directly associated with MyD88 and TLR4 [7] and TcpB targeted the Toll/IL-1 receptor domain-containing adaptor protein (TIRAP) dependent pathway by mimicking TIRAP's affinity towards phosphatidylinositol phosphate (PIP) resulting in inhibition of TLR2- and TLR4-mediated signaling [8, 9]. Another TIR domain containing protein Btp1 from* Brucella abortus* downmodulates maturation of infected dendritic cells by interfering with the TLR2 signaling pathway [10]. Very recently a TcpC homolog protein (TirS) in* Staphylococcus aureus* was shown to attenuate TLR-induced activation of NF-*κ*B and MAP-kinase signaling pathways* in vitro* and contribute to organ colonisation upon* S. aureus* infection* in vivo* [11]. The mechanism of bacteria interfering with host TLR signaling by Tcps, by blocking endogenous protein's association, was described as molecular mimicry [12].

We detected the sequence for a TIR domain containing protein in the genome of* Enterococcus faecalis* strain Symbioflor 1 (GenBank: HF558530.1, Gene ID: EFS1_1683) [13, 14]. The gene is also present in the published genomes of* E. faecalis* V583 (GenBank: AE016830.1, EF_1959) and* Enterococcus *sp. 7L76 draft genome (GenBank: FP929058). We assigned this gene the name* tcpF*, in accordance with the already described* tcpC* and* tcpB*. In this work we analyze the distribution of* tcpF* among clinical* E. faecalis* isolates, its genetic stability (allelic variation), the predicted protein structure, and its impact on the cellular innate response to infection. We report that* tcpF* was more frequent in* E. faecalis* isolates from urinary tract infections (UTIs) than in human faecal flora isolates. A* tcpF* deletion mutant of the model strain* E. faecalis* Symbioflor 1 induced elevated cytokine response upon infection of mouse macrophages compared to the parental wild-type strain and transient expression of TcpF in 293 human fibroblast cells led to suppression of the TLR2 triggered NF-*κ*B activation. Both findings indicate a role of TcpF in subversion of host defense by* E. faecalis* during infection.

## 2. Materials and Methods

### 2.1. Alignments and Protein Structure Calculation

Multiple protein sequence alignments were generated using Clustal Omega [15]. Protein tertiary structure prediction was conducted via* HHpred* by the Max-Planck Institute for Developmental Biology. Superposition of protein structures was done by TM-Align [16]. The structure of TLR1-TIR (1FYV) was downloaded from the protein data bank (http://www.rcsb.org/pdb/home/home.do). The presentations of the 3D TIR domain structures were accomplished by PyMOL software (http://www.pymol.org/).

### 2.2. Screening and Gene Sequencing

*E. faecalis* and* E. coli* isolation was performed from urine samples of patients with a clinically manifested UTI sent to the diagnostic unit of the Institute of Medical Microbiology, University Hospital Giessen and Marburg Ltd, branch Giessen. Colony PCR for tcps screening was done by picking one bacterial colony (ca. 10^5^ bacteria) and using the following primers:* E. coli*: UPECseq.1: 5′ GAATGTTTTGGGCAACAATATG-3′ and UPECseq.3: 5′-TCTTCTCCTGTATGCTATTTCAGC-3′;* E. faecalis*: E.f.seq.1 and E.f.seq.2 (Table 1). PCR was carried out for 30 cycles ((20 sec × 94°C/30 sec × 55°C/60 sec × 72°C) × 30/3 min × 72°C) with initial denaturation of 10 min × 94°C. An aliquot of the PCR was resolved on 0.8% agarose gel. The positive isolates were subjected to a second PCR reaction in order to obtain DNA for sequencing using the following primers: for* E. coli* sequencing: UPECseq.6: 5′-GTGGAAAACCTTCTATGCC-3′, UPECseq.7: 5′-GTGGTAGTTATCTATACCCTACATCTG-3′, and for* E. faecalis*: E.f.seq.3 and E.f.seq.5 (Table 1). Resulting PCR fragments were purified using the QIAquick PCR purification Kit (Qiagen). Sequencing was done by AGOWA genomics, Berlin (http://www.agowa.de/). The sequencing results were analyzed with* SeqManII* (DNAStar) software. A second set of 31* E. faecalis* isolates was collected from stool samples of non-UTI patients, where* E. faecalis* was determined to be not the cause of the illness, and screened for the presence of* tcpF* as described above. Statistical analysis for the significance of* tcpF* distribution among isolates was performed using Chi-squared test [6].

### 2.3. Bacterial Strains

*E. coli* CFT073 (NC004431) was a kind gift from U. Dobrindt, Institut für Molekulare Infektionsbiologie, JMU Würzburg, Germany.* E. faecalis* Symbioflor 1 (DSM 16431) is a commercially available probiotic strain and was provided by K. Zimmermann, SymbioPharm, Herborn, Germany [13].* E. faecalis* V583 was donated by J. Hübner, Division of Infectious Diseases, University Hospital Freiburg, Germany.* E. coli* CFT073 (UPEC) was grown on Columbia agar (Oxoid) or Luria-Bertani (LB) broth at 37°C.* E. faecalis* was grown in brain-heart-infusion (BHI) broth or on BHI agar plates (Difco) at 37°C.* E. coli* TOP 10 (Invitrogen) was grown in Luria-Bertani (LB) broth at 37°C on LB agar plates (Difco). If required, antibiotics were added with the following concentrations: ampicillin: 100 *μ*g/mL, kanamycin: 50 *μ*g/mL, and erythromycin: 10 *μ*g/mL for* E. faecalis *and 300 *μ*g/mL for* E. coli*. For infection assays* E. faecalis* were grown in BHI broth to early exponential phase (OD_600_ 0.1–0.5) and the bacterial concentration was calculated by optical density from a standardized growth curve.

### 2.4. DNA Isolation and Manipulation

Genomic DNA from* E. coli* and* E. faecalis* was isolated using the Genomic DNA purification kit (Gentra). Plasmid preparations from* E. coli* TOP 10 were done using the QIAfilter Plasmid Midi Kit (Qiagen). Knock-out of the* tcpF* gene in the genome of* E. faecalis* Symbioflor1 was done as described [17]. Briefly, the flanking regions of the* tcpF* gene were amplified by PCR (Expand High fidelity PCR system, Roche) using the primer pairs Ef-Del-1A 5′-AGCACTGGATCCGAACCAATTCTCCTTATACTCG-3′ and Ef-Del-2 5′-GACGACTACACAGTGCTCACTACTCAACATCCTCCA-3′, giving rise to a 345-bp product I, Ef-Del-3 5′-ACTGTGTAGTCGTCGGTAGAGTAATTGGAGGAGGT-3′, and Ef-Del-4 5′-AGCAGTGAATTCAATGCAGCTGTTTCTAACAACCGA-3′, generating a 405-bp product II. A second PCR was performed with primers Ef-Del-1A and Ef-Del-4 using both products I and II as a template to amplify a 736-bp DNA fragment, which was subsequently digested (*Bam*HI and* Eco*RI), ligated into the temperature sensitive shuttle vector pAULA (25), and transformed into* E. coli *TOP10. The vector construct was isolated, sequenced, and electroporated into* E. faecalis *to achieve gene deletion.

*TcpF *and* tcpC* were cloned into the eukaryotic expression plasmid pCMV-Flag-2B (Stratagene), generating the plasmids pSty10 (TcpF) and pSty13 (TcpC1). The following primer combinations were used to amplify genomic DNA for the cloning: TK9: 5′-ACTACTGGTCTCAGCGCCAATGAGAGTATCAGT-3′ and TK10: 5′-ACGACTGGTCTCATATCAGATAAATAATTTAACTGGCT-3′ for* E. faecalis TcpF *(pSty10); pTcpC-Flag For: 5′-ATCCGCGGATCCATAGCATATGAAAACATAGAATTT-3′ and pTcpC-Flag Rev: 5′-GCGAGTCTCGAGTTATCTTCTCCTGTATGCTATTTCAGC-3′ [6] for full length* E. coli tcpC (TcpC1) *originally a GTG start codon (pSty13). The vector and the amplified DNA fragments were cut with* Bam*HI and* Xho*I (Fermentas) in order to prepare compatible cohesive ends for ligation.

### 2.5. RNA Isolation and RT-PCR

For RNA isolation,* E. faecalis *Symbioflor 1 and V583 were harvested after growth in BHI broth or from the cell culture supernatants (DMEM) of CaCo cells after 1, 2, or 3 h of infection. Total RNA was isolated using the RNeasy Protect Bacteria Mini Kit (Qiagen) following the manufacturers' protocol with lysozyme lysis. The RNA was eluted in RNase free water, digested with 2 U of Turbo DNaseI (Ambion) for 30 min at 37°C, and again purified using the RNeasy Mini Kit (Qiagen). RNA was recovered in RNase free water, heat denatured for 10 min at 65°C, and quantified with the NanoDrop ND-1000 UV-Vis Spectrophotometer (NanoDrop Technologies), and a quality profile with the Agilent 2100 bioanalyzer (Agilent Technologies) was made. For RT-PCR, 400 ng total RNA was reverse transcribed using 50 U Superscript II Reverse Transcriptase (Life Technologies) in a 20 *μ*L reaction using primers E.f.seq. 7 or E.f.seq. 2 (Table 1) following the instructions of the manufacturer. PCR was done with 3 *μ*L of first strand cDNA in a 30 *μ*L reaction using 1 U Taq Polymerase (Life Technologies). PCR was carried out for 30 cycles ((20 sec × 94°C/30 sec × 53–56°C/60 sec × 72°C) × 30/3 min × 72°C). 8 *μ*L of the PCR reaction was resolved on 1.5% agarose gels.

### 2.6. Luciferase Reporter Assay

Human TLR2 and luciferase expressing plasmids are described previously [18]. Confluent overnight cultures of HEK 293 cells in 12-well plates, grown in DMEM (Gibco) supplemented with 10% foetal calf serum (PAA Laboratories) and 1x penicillin/streptomycin (Gibco), were washed in DMEM and transfected with a mixture of 2.4 *μ*g plasmid DNA and 7 *μ*L Lipofectamine 2000 (Invitrogen). All assays were performed with a constant amount of reporter constructs as follows: 550 ng pELAM-Luc and 50 ng phRL-TK (Promega), 50 ng phTLR2, and increasing amounts of TcpC1 (50 ng to 1550 ng of pSty13) or TcpF (50 ng to 1550 ng of pSty10). DNA to Lipofectamine ratio was kept stable by using the empty CMV-promoter vector pRK5 (BD Pharmingen). After 6 h the medium was replaced with DMEM supplemented with 10% FCS and after an additional 2 h, the cells were split, transferred to 96-well plates at 4 × 10^4^/well, and incubated overnight in DMEM supplemented with 5% FCS. On the next day the cells were stimulated with 0.5 *μ*M Pam_3_Cys-SKKKK × 3 HCl (EMC microcollections GmbH, Tübingen) in DMEM. Control samples were treated with an equal volume of DMEM. Stimulated cells were incubated for 5 h, lysed afterwards with 20 *μ*L Passive Lysis Buffer (Promega), and stored at −20°C. Measurement of NF-*κ*B mediated luciferase activity was carried out with the Dual-Luciferase Reporter Assay System (Promega) using an L-max plate reader (Molecular Devices). Signal measurements were performed in triplicate and experiments were repeated at least three times. The primary firefly luciferase signal was normalized to the signal of* Renilla* luciferase in each well, resulting in relative luciferase activity. Results are presented as fold changes of stimulated to nonstimulated activity.

### 2.7. Cytokine Assays

RAW 264.7 mouse macrophages were seeded on 24-well plates at 6 × 10^5^ cells/well in 500 *μ*L DMEM supplemented with 10% FCS and 1x penicillin/streptomycin. On the next day the cells were washed twice with PBS (37°C) and covered with 400 *μ*L DMEM + 1% FCS. The RAW 264.7 cells of two control wells were harvested and counted in order to define exact cell numbers. 100 *μ*L bacterial suspension in DMEM, containing a defined CFU number, was added to the cells and incubated for 5 h. Supernatants were collected from cells infected with* E. faecalis *Symbioflor 1 and the isogenic* ΔtcpF *mutant. The secretion of TNF-*α* was measured using the mouse TNF-*α* Quantikine ELISA kit (BD) on a Phomo Plate reader (Anthos Mikrosystme) according to the kits manual.

## 3. Results

### 3.1. *In Silico* Modeling of TcpF

In a database search of bacterial genomes using* conserved domain database* (CDD) on NCBI, we identified a gene encoding for a TIR domain containing protein in the genome of* E. faecalis *strain Symbioflor 1 (Gene ID: EFS1_1683). The sequence spans 825 base pairs coding for a 274-amino-acid-long protein with predicted mass of 32650 Da. Concordant with previous studies [6], we assigned this gene the name* TIR domain containing protein of E. faecalis* (*tcpF*). Additionally, a multiple primary sequence alignment of the human TIR domain containing proteins involved in TLR2 signaling, TLR1, TLR2, MyD88, and MAL, and the bacterial TIR domain containing proteins PdTLP, TcpC, and TcpF [12] revealed strong consensus in the Box1 motif [19], the last two residues, proline (P) and glycine (G), of the Box2 motif, and the residues forming the *β*C-strand and the *α*C-helix (Supplementary Figure 1 in Supplementary Material available online at http://dx.doi.org/10.1155/2014/918143). The primary sequence of TcpF-TIR exhibits common TIR domain motifs. The predicted tertiary structure of TcpF-TIR shows a typical TIR domain fold with parallel *β*-strands surrounded by *α*-helices and loops (Supplementary Figure 2(a)* In silico* structure prediction of TcpF-TIR reveals structural similarity to TLR1-TIR.). The TIR domain of TcpF is located at the N-terminus and consists of 4 *β*-strands, which are coated by 4 *α*-helices. The superposition of TcpF-TIR and TLR1-TIR reveals considerable similarity between those two structures (Supplementary Figure 2(b)). In particular, strong similarity can be observed at the BB-loop, which has been reported to be part of the interface of TLR1-TIR and TLR2-TIR interaction (Supplementary Figure 2(c)) [20]. Also located at the C-terminus of TcpF, we found the motif KVRFKLKK similar to a functional motif in TcpB of* Brucella*, a PIP2 binding domain with the basic amino acid sequence KKRxxxxKK. It was reported that this motif promotes colocalization with the plasma membrane of the eukaryotic cell by binding to membrane phosphoinositides [9].

### 3.2. Screening of* E. faecalis* Strains for* tcpF*

**Figure 1:**
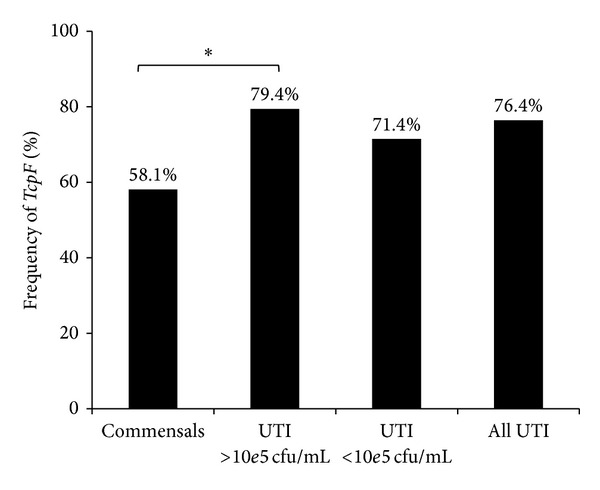
*TcpF* frequency among UTI and commensal isolates.* TcpF* is associated with cfu > 10e5/mL upon infection of the urinary tract by* E*.* faecalis*.* TcpF* was detected by PCR. Commensals *n* = 31, UTI > 10e5cfu/mL, *n* = 68 (∗*P* = 0,028), UTI < 10e5cfu/mL *n* = 42. Statistical analysis was performed using Chi-squared test.

A total of 110 urine isolates of patients with clinically diagnosed UTI and 31 faecal flora* E. faecalis* isolates were screened via PCR with primers E.f.seq.1 and E.f.seq.2 positioned at the start and the end of the* tcpF* open reading frame (ORF). The UTI isolates were collected in a period of three months from samples sent to the microbiology diagnostic lab of the Institute of Medical Microbiology. The samples originate from patients admitted to the University Hospital in Giessen with clinically apparent UTI. Among UTI samples,* E. faecalis* colony-forming unit (CFU) numbers varied with respect to the threshold of <10^5^/mL and >10^5^/mL; the latter fulfill the microbiological definition of UTI.* E. faecalis* stool isolates were derived from stool samples of non-UTI patients, where* E. faecalis *was determined as not the causative agent, but element of the stool commensal flora.* TcpF* was found in 76.4% of all UTI isolates. Interestingly,* tcpF* was present in 79.4% of UTI isolates from samples with CFU numbers > 10^5^/mL but was less common in UTI isolates from samples with CFU numbers < 10^5^/mL (71.4%) or isolates from faecal flora (58.1%) (Figure 1). These numbers suggest that the presence of* tcpF* benefits the colonization of the human urinary tract (UT) and promotes* E. faecalis* survival.

### 3.3. Genetic Analysis of* tcpF*

**Figure 2:**
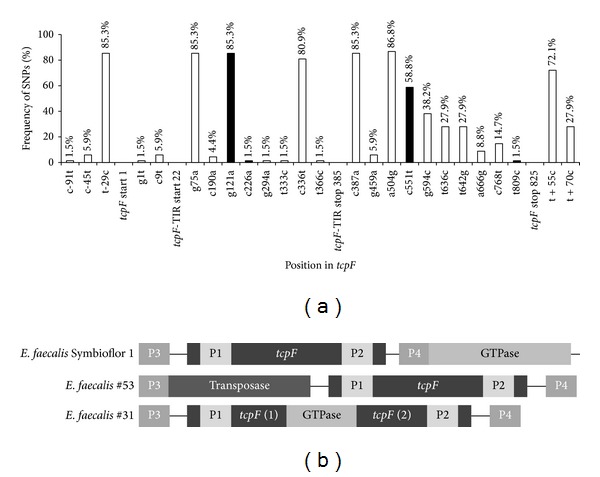
*TcpF'*s sequence polymorphism. (a) SNP frequency in the* tcpF *locus of 68* E. faecalis* UTI isolates. Sequence alignment was conducted on* tcpF* of* E. faecalis* Symbioflor 1. Black bars indicate mutations resulting in amino acid substitutions and white bars indicate silent mutations. The amino acid substitutions are G121A: Ala-Thr, C226G: Gln-Lys, C551T: Thr-Ile, and T809C: Leu-Ser. (b) Genetic structure of the* tcpF* locus of* E. faecalis *isolates. Two major rearrangements were observed in the UTI isolates* E*.* faecalis* #53 and #31. P1/P2: primer positions for the screening-PCR; P3/P4: primer positions for the sequencing-PCR.

We further examined the genetic stability of* tcpF* among urine isolates by sequencing the target gene and the neighboring regions. A PCR with primers E.f.seq.3 and E.f.seq.5 was done for a total of 68 isolates including isolates from urine samples with more than 10^5^ CFU/mL (*n* = 44) and less than 10^5^ CFU/mL (*n* = 22). The primers are located about 150–200 bp up- and downstream from the* tcpF* gene. The amplified PCR fragment has an expected length of 1206 bp. Primers E.f.seq.3 and E.f.seq.5 were reused for bidirectional sequencing. The SNPs aligned to the* tcpF* sequence of* E. faecalis* Symbioflor 1 are shown in Figure 2(a). In total, we identified 18* tcpF* genotypes and found four nucleotide substitutions leading to amino acid changes. The transition G121A was the most frequent (85.3%); thus it seems to be the more stable variant. This SNP is located within the TIR domain and leads to the change of alanine to threonine. The transversion C226G is located also within the TIR domain resulting in a glutamine to lysine exchange. Only one strain showed this substitution. The transitions C551T and T809C are outside of the TIR domain. C551T results in a threonine to isoleucine exchange and was found in 40* tcpF* positive isolates (58.8%). T809C was found in one isolate and results in leucine to serine exchange. The G121A and C551Talleles were randomly distributed among the isolates from urine samples with CFU > 10^5^/mL and CFU < 10^5^/mL.

Two* E. faecalis* urine isolates produced PCR fragments of unexpected length suggesting a larger insertion within the primers' positions (Figure 2(b)). Isolate* E.f.#*53 yielded the expected fragment of 844 bp in the screening PCR. However, the sequencing PCR produced a fragment of about 2000 bp by expected 1206 bp. The sequencing revealed a 5′end insertion followed by the open reading frame of* tcpF*. The search in the genome of* E. faecalis* Symbioflor 1 showed this inserted sequence to encode for a gene with annotated transposase function (EFS1_1430). Isolate* E.f.#*31 produced a fragment of unexpected length in the screening as well as in the sequencing PCR. The amplified fragment of the screening PCR was about 1800 bp and the fragment of the sequencing PCR was about 2100 bp, revealing that the ORF of* tcpF* was disrupted by an inserted fragment. A genomic BLAST search identified a homolog sequence in the genome of* E. faecalis* V538 encoding for the gene downstream of* tcpF* with annotated putative deoxyguanosine triphosphate triphosphohydrolase (GTPase) function (EF_1958). In both, the genomes of* E. faecalis* Symbioflor 1 and V538, the putative GTPase gene starts in close proximity (17 bp) to the 3′end of* tcpF*.

In order to compare* tcpF*'s genetic variation to other bacterial* tcps* we screened 100* E. coli* isolates derived from urine specimens of UTI patients for the presence of the* tcpC* sequence. All isolates originated from samples with CFU numbers > 10^5^/mL. 20* E. coli *isolates (20%) were* tcpC* positive. Unlike* tcpF*, sequencing of* tcpC* revealed very low genetic polymorphism. The sequence of 19 isolates was identical to CFT073's* tcpC*. One isolate only showed two point mutations. The transition G385A led to an alanine to valine exchange, whereas the transition C482T was silent.

**Figure 3:**
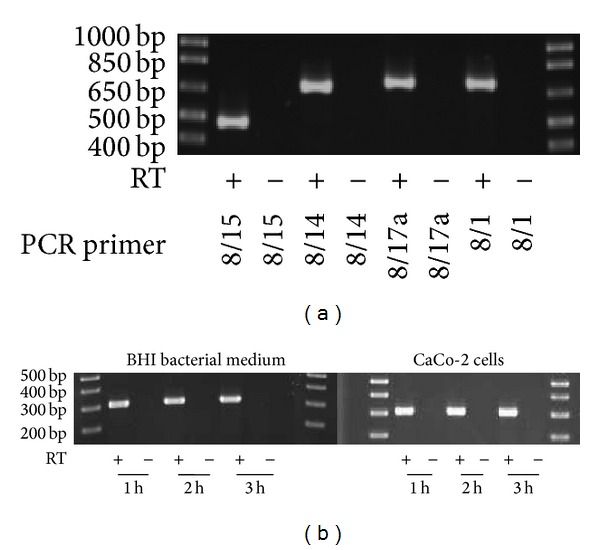
*TcpF* is constitutively transcribed in* E*.* faecalis* Symbioflor 1. (a) Full length mRNA transcript of* tcpF* in* E*.* faecalis* Symbioflor 1. Reverse transcription was performed with primer E.f.seq.2. PCR primers E.f.seq. 8/15: 457 bp, primers E.f.seq. 8/14: 640 bp, primers E.f.seq. 8/17a: 671 bp, and primers E.f.seq. 8/1: 680 bp. (b)* TcpF* mRNA is transcribed upon growth in BHI medium and upon infection on CaCo cells. PCR primers E.f.seq. 7/6: 300 bp. Reverse transcriptase minus (RT-) reactions served as negative control.

The transcription of* tcpF* mRNA was verified for* E*.* faecalis* Symbioflor 1 (Figure 3(a)) as well as for the pathogenic isolate V583 (data not shown). cDNA transcripts with increasing length were observed using reverse transcription primer E.f.seq.2 positioned between base pairs 809 and 832 (stop codon TAA at 823–825 bp), and PCR with primers which span the length of the gene up to the start codon.* TcpF *was transcribed during growth of bacteria in BHI medium and also by coincubation (infection) with colon carcinoma CaCo-2 cells for up to three hours (Figure 3(b)).

### 3.4. Functional Analysis of TcpF

**Figure 4:**
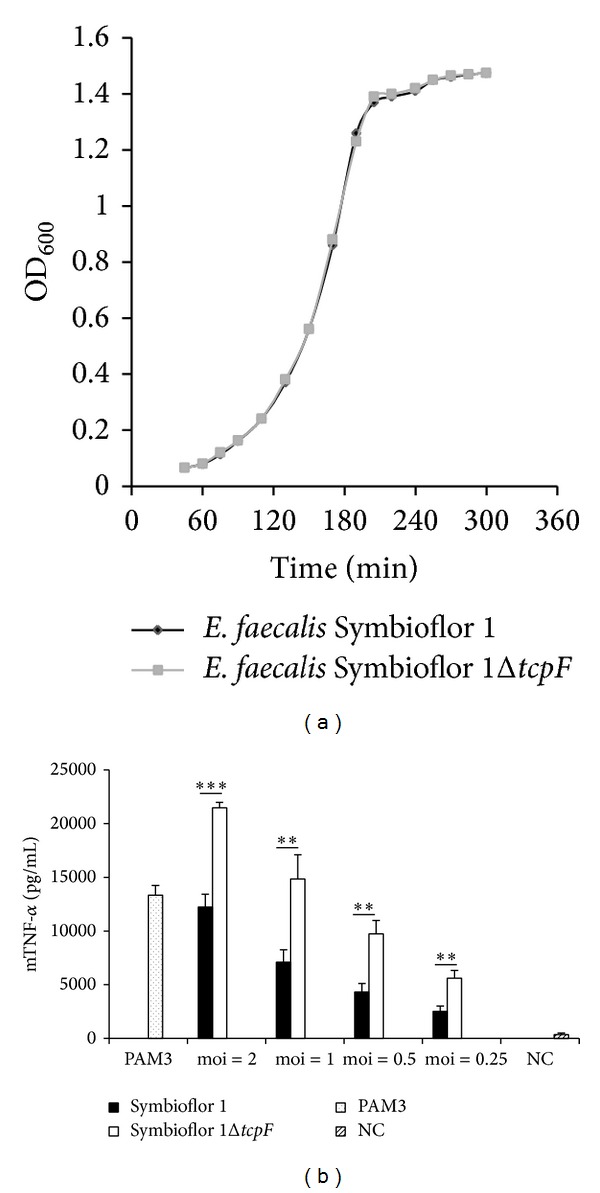
TcpF suppresses TNF-*α* induction. (a)* E. faecalis *Symbioflor 1 and its isogenic* tcpF* deletion mutant Symbioflor 1 Δ*tcpF *show identical growth rate. Fresh culture was started from overnight culture 1 : 25 in BHI at 37°C and 100 rpm. Optical density at 600 nm (OD_600_) at indicated time points. (b) RAW264.7 macrophages were infected for 6 h with* E. faecalis *Symbioflor 1 and* E*.* faecalis* Symbioflor 1 Δ*tcpF* at indicated moi. NC: negative control—uninfected macrophages. Error bars represent s.d. (*n* = 4). Student's *t*-test: ∗∗∗*P* < 0,001; ∗∗*P* < 0,01.

**Figure 5:**
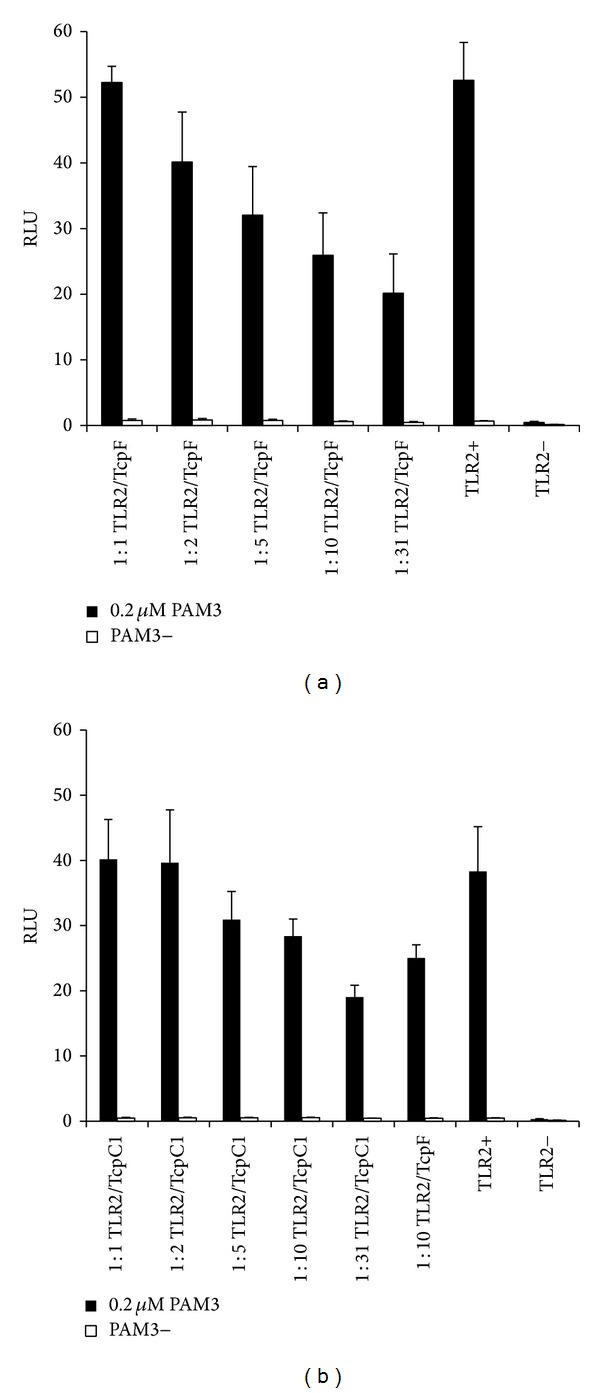
Intracellular expression of TcpF impairs TLR2 dependent NF*κ*B activation. HEK293 cells were transiently transfected with hTLR2 (50 ng), pRK5, reporter plasmids pELAM-Luc and phRL-TK, and increasing amounts of TcpF (a) or TcpC1 (b) expressing plasmids at ratios of 1 : 1, 1 : 2, 1 : 5, 1 : 10, and 1 : 31. Cells were stimulated with 0.2 *μ*M PAM3 and incubated for 5 h. Nonstimulated and TLR2 deficient cells (TLR2-) served as controls. Error bars represent s.d. (*n* = 3). RLU: relative luciferase unit.

In order to analyze the function of TcpF during infection, we created a* tcpF* deletion mutant of the model strain* E. faecalis* Symbioflor 1 = Symbioflor 1 Δ*tcpF*. The deletion did not affect bacterial growth in BHI medium (Figure 4(a)), but infection of mouse RAW 264.7 macrophages with the deletion mutant led to significantly higher TNF-*α* secretion in medium than that caused by the wild-type strain* Symbioflor* 1 (Figure 4(b)), revealing inhibition of stress signaling by TcpF. Further studies should include infection experiments of mouse macrophages with a complemented strain (*E. faecalis* Symbioflor Δ*tcpF* +* tcpF*) in order to additionally support and verify the knock-out phenotype and gene function. Previous studies have shown that immune suppression by bacterial Tcps is based on direct interaction with host's Tcps leading to interference with TLR signaling [6, 7, 11]. TLR2 is responsible for sensing Gram-positive pathogens [18]; hence we expressed cloned TcpF and TcpC in human fibroblast cells and monitored the effect on the TLR2 dependent NF-*κ*B activation. Therefore, HEK293 cells were transiently transfected with plasmids expressing the firefly luciferase gene under the control of an NF-*κ*B dependent promoter (pELAM-Luc) and plasmids constitutively expressing human TLR2 (phTLR2). Plasmids for constitutive eukaryotic expression of Tcps : pSty10 (TcpF) or pSty13 (TcpC of UPEC CFT073) were cotransfected in a ratio of 1 : 1, 1 : 2, 1 : 5, 1 : 10, and 1 : 31 to phTLR2. Transfected cells were stimulated with Pam_3,_ a synthetic ligand of TLR2. Intracellular expression of TcpF resulted in dose dependent NF-*κ*B signal suppression comparable to that of TcpC (Figure 5). These results suggest that TcpF interferes with TLR signaling in a similar manner to TcpC [6] resulting in markedly reduced cytokine release and impaired response upon infection.

## 4. Discussion

Over the last decade several bacterial TIR domain-containing proteins (Tcps) have been identified [21], yet all but one were found in Gram-negative bacteria [11]. Using a conserved protein domain search we discovered a homologous sequence for the TIR domain encoded by a gene of previously unknown function in the genome of* E. faecalis *(*E. faecalis* V583: EF_1959;* E. faecalis* Symbioflor 1: EFS1_1683) [13, 14].* Enterococci* have until recently been considered harmless or even beneficial commensals of the gastrointestinal tract, the oral cavity, and vagina in humans. However, there is growing evidence that these bacteria possess specific traits that enable them to colonize new habitats in the host and cause infections such as bacteremia, peritonitis, endocarditis, device-related infections, and urinary tract and wound infections [22].* Enterococci*, and particularly the species* E. faecalis*, are second to* E. coli* to cause UTIs [23].

In a screening of UTI and stool isolates, we found that the TIR containing gene,* tcpF*, is less frequent in commensal* E. faecalis* strains (58.1%) than in isolates from UTI (76.4%) and this difference becomes more significant when only the UTI isolates with cfu numbers higher than 10e5/mL (79.4%* tcpF* positive) are compared with the commensals (*P* = 0.028) (Figure 1), suggesting that the* tcpF* promotes bacterial survival and therefore constitutes a selective advantage for the colonization of the human UT by* E. faecalis*. Similar observations were made by Cirl et al. 2008 [6] regarding the TIR domain containing protein of* E. coli* (TcpC). They reported that TcpC promotes intracellular survival in immune and epithelial cells and that* tcpC* was associated with severity of UTI in humans. In their study* tcpC *was found in 40% of acute pyelonephritis isolates but was less common in cystitis (21%), asymptomatic bacteriuria (16%), or commensal (8%)* E. coli* strains. Interestingly, we found* tcpF* to be also abundant (58.1%) among commensal (nonpathogenic) isolates from faecal flora, indicating a stronger beneficial effect of the Tcp gene for* E. faecalis* than that for* E. coli* (8%) in the colonization of the human GI tract.

The sequence analysis of* tcpC* revealed just one point mutation with a subsequent amino acid change and one silent point mutation among 20* tcpC* positive* E. coli* isolates. Both nucleotide substitutions do not concern the TIR domain. However, in the 825 bp long sequence of* tcpF* we detected point mutations in 20 positions and 4 of them resulted in subsequent amino acid changes. Two of them are within the TIR domain, yet one of them C226G is of very low frequency (1 in 68). Thus, in contrast to* tcpC, tcpF* shows a moderate genetic polymorphism, which however translates only to a lower extent in primary amino acid sequence variation.* In silico* modeling of the TIR domain of TcpF reveals a conserved (stable) amino acid sequence resulting in conserved secondary and tertiary structures (Supplementary Figure 2(a)). The observed alteration Ala41Thr leads to structural changes in the beginning of the BB-loop (Supplementary Figure 2(d)), whereas the alteration Gln76Lys does not influence the TIR domain structure (Supplementary Figure 2(e)). Whether Ala41Thr affects binding towards possible binding partners is a matter of speculation, which requires further experiments, for example, TLR2 coexpression, to provide a definite answer. Remarkably, we observed also two major insertions in this gene locus among the isolated strains. In the case of* E.f.*#53 a transposition led to alteration of the neighboring sequence of* tcpF*. In the case of* E.f.*#31, an insertion of the downstream positioned GTPase gene into the* tcpF* ORF took place, most probably the result of an inversion (Figure 2(b)). It appears that the GTPase (EFS1_1682; EF_1958) was part of the insertion loop and was rearranged into the* tcpF* gene, based on the known insertion mechanisms [24]. Both locus rearrangements and also the higher number of single nucleotide polymorphisms in* tcpF* compared to* tcpC* support the hypothesis of a process of ongoing selection and a more recent acquisition of the sequence in the genome of* E. faecalis*. Interestingly, in all* tcpF* positive isolates* tcpF* is always paired with the downstream positioned putative GTPase gene. However, no functional dependency or interrelationship between both genes has been reported yet or was predicted when comparing homologous protein domains in other bacteria. Both genes are not part of any known cluster being positioned 350 bp away from the next upstream and 630 bp from the next downstream neighbor.

Bacterial Tcps have been postulated to modulate host immune response by interference with the TLR signaling pathway [11, 12]. Using the mouse macrophage line RAW264.7 to investigate the effect of TcpF on host immune cells, we found that infection with the* tcpF* deletion mutant of* E. faecalis *strainSymbioflor 1 led to significantly higher TNF-*α* response as compared to wild-type (Figure 4(b)). TNF-*α* promotes the acute phase reaction and leads to the recruitment of inflammatory cells [4]. Our results show that TcpF enables* E. faecalis* to attenuate host's cytokine response upon infection to avoid elimination by immune cells. Finally, the ability to suppress the TLR2 dependent NF-*κ*B activation for TcpF was shown to be similar to the suppression caused by TcpC as demonstrated by Cirl et al. 2008 [6]. TLR2's main ligands are bacterial lipoproteins allowing the receptor to react by stimuli from both Gram-positive and Gram-negative bacteria [3]. The intracellular expression of TcpF suppressed the TLR2 dependent NF-*κ*B signal in a dose dependent manner (Figure 5). Considering molecular mimicry of human TIR domains as the mechanism for the immune modulatory effect of bacterial TIR containing domain proteins, TcpF's possible targets in the TLR2 dependent pathway are the TIR domains of TLR2, TLR1, TLR6, and MyD88 as well as MAL [4]. The structure prediction revealed that TcpF-TIR shows high similarity to TLR1-TIR. Since the BB-loop of TLR1-TIR and in particular the Gly 49 within the BB-loop (Supplementary Figure 2(c)) were shown to be involved in TLR1-TIR and TLR2-TIR interaction [20], it seems likely that TcpF may compete with the TIR domain of human TLR1 for TLR dimer formation and so abolishes further downstream signaling. In this context a colocalization of TcpF with the cytoplasmic part of TLRs may be facilitated by the presence of the KVRFKLKK motif at the C-terminus of TcpF as reported for the KKRxxxxKK motif in TcpB of* Brucella* [9].

In spite of their pathogenic potential, enterococci are exemplary commensals as evidenced by their presence as natural colonizers of the GI tract and by the fact that they have been used safely for decades as probiotics [13, 25]. Avoiding activation of NF-*κ*B via TLR inhibition is crucial for homeostasis in the GI tract, where epithelial cells are in constant contact with the commensal microbiota. Accordingly, inhibitory molecules that minimize the risk of perpetual activation, such as the TLR inhibitors PPARy, A20, NOD2, IRAK-M, SIGIRR, and Tollip, are essential for sustaining mucosal homeostasis in the human gut [26]. Inappropriate activation of TLR signaling pathways leads to deleterious inflammation and has been implicated in the pathogenesis of GI disorders, including inflammatory bowel disease [27] and colon cancer [28]. On account of the higher frequency of polymorphisms detected in the* tcpF* ORF, this gene's acquisition appears to be a relatively recent event for the genome of* E. faecalis*. This gene seems to be beneficial for the colonization of the UTI tract by* Enterococcus faecalis* since 79% of UTI isolates with CFU numbers > 10^5^/mL carry this gene and even 71.4% of the UTI isolates with CFU numbers < 10^5^/mL are also positive for the gene. On the other hand, the gene is also present in more than the half of commensal isolates from stool samples; thus this recently acquired gene is spreading successfully among commensal* E. faecalis* strains, also indicating a positive effect for the colonizing bacteria in the gut. In both the pathogenic and commensal strains, the presence of* tcpF* serves the paradigm of attenuating TLR2 signaling, therefore limiting bacterial recognition and immune activation by the host and therefore enabling colonizing bacteria to reach higher cfu numbers. It appears that TcpF promotes* E. faecalis* invasion and survival in the UTI and while in its natural polymicrobial habitat, the human GI tract, TcpF benefits the intestinal homeostasis.

## 5. Conclusion

In this work we provide evidence that TcpF from* E. faecalis* is a new member of the family of bacterial Tcps in the phylum of Gram-positive bacteria.* In silico *structure prediction of TcpF-TIR showed a characteristic TIR domain fold and strong similarity to TLR1-TIR in particular. A screening of UTI isolates of* E. faecalis* revealed that* tcpF* is more frequent among isolates of human urinary tract infections than stool flora, particularly in isolates with bacterial counts of more than 10e5 cfu/mL (*P* = 0.028).* In vitro*, TcpF was shown to impair the secretion of proinflammatory cytokines during infection of host cells and to interfere with TLR2 signaling upon intracellular expression, supporting the concept of molecular mimicry, as imposed to this group of proteins. Due to TcpF's ability to negatively modulate recognition by the TLR2 receptor and NF-*κ*B activation and the fact that* tcpF* not only is common among* E. faecalis* isolates from intestinal flora but even prevails among human UTI isolates (Figure 1), it appears that* tcpF* represents a recently acquired adaptive feature for* E. faecalis* that favors UT invasion and generally benefits mucosa colonization.

## Supplementary Material

Supplementary Figure 1: The primary sequence of TcpF-TIR exhibits common TIR domain motifs. Primary sequence alignment of human Tcps involved in TLR2 signaling and bacterial Tcps of Paracoccus denitrificans (PdTIR), Escherichia coli (TcpC-TIR) and Enterococcus faecalis (TcpF-TIR). The regions corresponding to the conserved TIR motifs, boxes 1,2 and 3 are indicated by red, blue and yellow rectangles, respectively. Secondary structure elements of TLR1-TIR (PDB: 1FYV) are shown above the alignment. The alignment was generated using ClustalOmega.
Supplementary Figure 2: In silico structure prediction of TcpF-TIR reveals structural similarity to TLR1-TIR. (A) Predicted tertiary-structure of TcpF-TIR (residues 7-128). According to previous studies *α*- helices are labeled as *α*(A-B) and *β*-pleated sheets as *β*(A-B). (B) Superposition of TcpF-TIR and TLR1-TIR (PDB: 1FYV). TcpF-TIR is colored in magenta and TLR1-TIR in green. Secondary structure elements of TcpF-TIR are labeled as previously described. The BB-loop refers to the connecting loop between the second *β*-pleated sheet (*β*B) and the second *α*-helix (*α*B). (C) Structural alignment of the BB-loops of TcpF-TIR and TLR1-TIR. TcpF-TIR is colored in magenta and TLR1-TIR in green. Residue positions refer to sequences of TcpF and TLR1.Protein tertiary-structure prediction based on homology detection was performed using HHpred by the Max-Planck Institute for Developmental Biology. Structural alignment was generated using TM-align by Zhang-Lab and illustration was composed using pymol (Schroedinger). (D) Superposition of TcpF-TIR (magenta) and its Ala41Thr (blue) mutant. (E) Superposition of TcpF-TIR (magenta) and its Gln76Lys (blue) mutant.

## Figures and Tables

**Table 1:** RT and PCR primer for *Enterococcus faecalis*.

Primer	Sequence	5′ position
E.f.seq.1	5′-GATGTTGAGTAGTGAGCAACG-3′	−12 s
E.f.seq.2	5′-CTCCTCCAATTACTCTACCTTCTC-3′	832 as
E.f.seq.3	5′-CCGTACATCGCTCTATAATCAC-3′	−203 s
E.f.seq.5	5′-GTCTAAATGCATCACTTTTAATTACTC-3′	995 as
E.f.seq.6	5′-AGCCATTCCAAAATAAACTGAC-3′	470 s
E.f.seq.7	5′-GAATTCCCGATTGTTATACG-3′	770 as
E.f.seq.8	5′-GCTTTCATCCTCTGATAGCGT-3′	606 as
E.f.seq.14	5′-AGTCATAGTTCAAAGGATCAAG-3′	29 s
E.f.seq.15	5′-CTGATCATTATTATCAGAGTATTCCATG-3′	212 s
E.f.seq.17a	5′-GTAGTGAGCAACGGGAAAAAGAT-3′	−3 s

s: sense.

as: antisense.
