# Supplementary figures and images for: The TIR Domain Containing Locus of Enterococcus faecalis Is Predominant among Urinary Tract Infection Isolates and Downregulates Host Inflammatory Response
Source: Int J Microbiol. 2014 Jul 24;2014:918143. doi: 10.1155/2014/918143 (PMC4131471; doi:10.1155/2014/918143)

## Slide 1
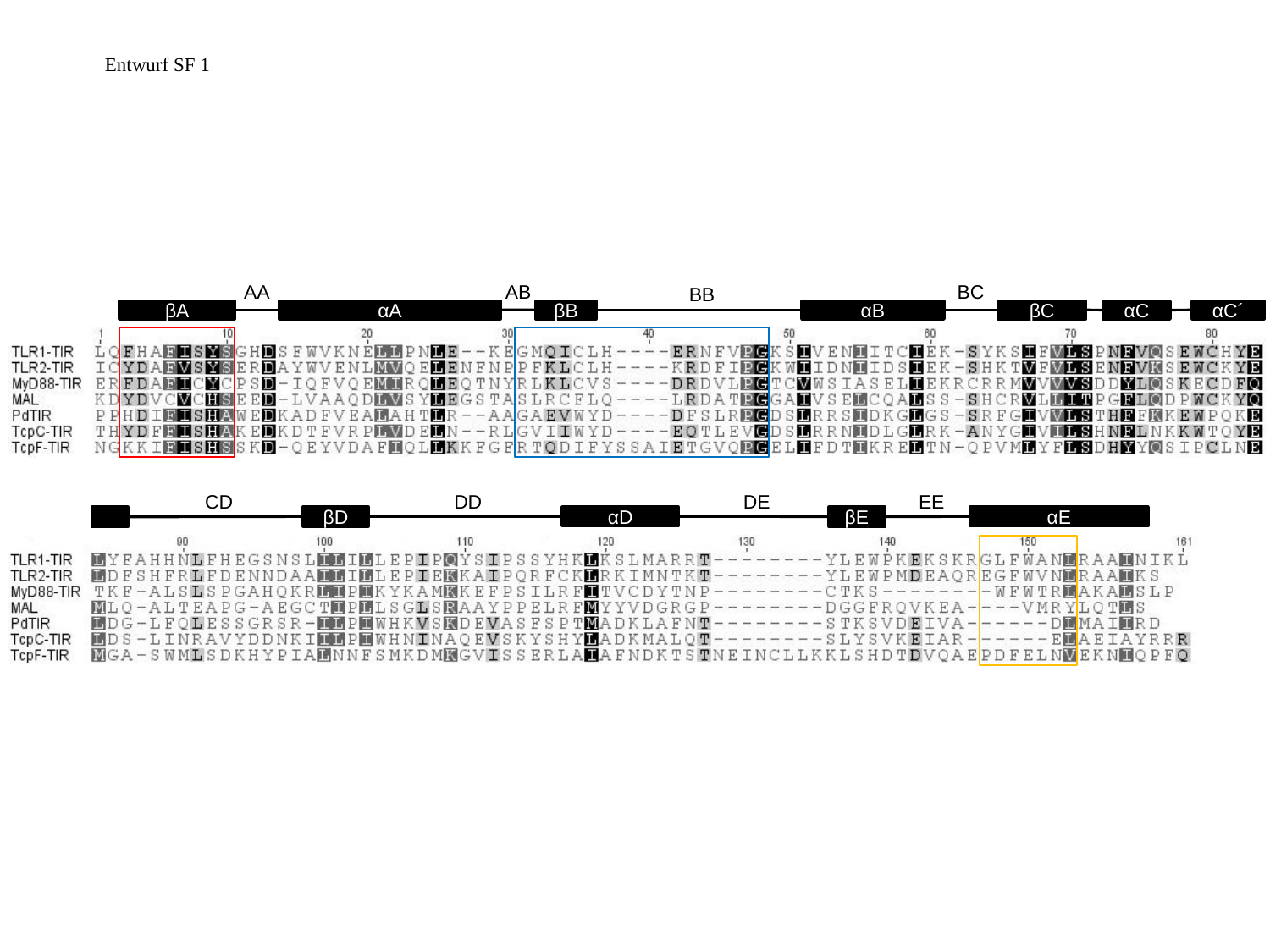

Entwurf SF 1
AA
AB
BC
BB
βA
αA
βB
βC
αB
αC
αC´
CD
DD
EE
DE
βD
αD
βE
αE

Supplement: Supplementary file 1 — Supplementary Figure 1: The primary sequence of TcpF-TIR exhibits common TIR domain motifs. Primary sequence alignment of human Tcps involved in TLR2 signaling and bacterial Tcps of Paracoccus denitrificans (PdTIR), Escherichia coli (TcpC-TIR) and Enterococcus faecalis (TcpF-TIR). The regions corresponding to the conserved TIR motifs, boxes 1,2 and 3 are indicated by red, blue and yellow rectangles, respectively. Secondary structure elements of TLR1-TIR (PDB: 1FYV) are shown above the alignment. The alignment was generated using ClustalOmega. Supplementary Figure 2: In silico structure prediction of TcpF-TIR reveals structural similarity to TLR1-TIR. (A) Predicted tertiary-structure of TcpF-TIR (residues 7-128). According to previous studies α- helices are labeled as α(A-B) and β-pleated sheets as β(A-B). (B) Superposition of TcpF-TIR and TLR1-TIR (PDB: 1FYV). TcpF-TIR is colored in magenta and TLR1-TIR in green. Secondary structure elements of TcpF-TIR are labeled as previously described. The BB-loop refers to the connecting loop between the second β-pleated sheet (βB) and the second α-helix (αB). (C) Structural alignment of the BB-loops of TcpF-TIR and TLR1-TIR. TcpF-TIR is colored in magenta and TLR1-TIR in green. Residue positions refer to sequences of TcpF and TLR1.Protein tertiary-structure prediction based on homology detection was performed using HHpred by the Max-Planck Institute for Developmental Biology. Structural alignment was generated using TM-align by Zhang-Lab and illustration was composed using pymol (Schroedinger). (D) Superposition of TcpF-TIR (magenta) and its Ala41Thr (blue) mutant. (E) Superposition of TcpF-TIR (magenta) and its Gln76Lys (blue) mutant. [file 918143.f1.zip › 1017134.pptx]
